# Prevalence of Cervical Dysplasia in HIV-Positive and HIV-Negative Women at the Sihanouk Hospital Center of HOPE, Phnom Penh , Cambodia

**DOI:** 10.31557/APJCP.2019.20.2.653

**Published:** 2019

**Authors:** Sovannara Thay, Sally Ann Peprah, Chin Hur, Angela C Tramontano, Ellen Maling, Andrew T Goldstein, Christina Hong

**Affiliations:** 1 *Sihanouk Hospital Center of HOPE, *; 6 *Lūcerent Clinical Solutions, Cambodia, *; 2 *Johns Hopkins University,*; 3 *Harvard Medical School,*; 4 *Institute for Technology Assessment,*; 5 *Department of Obstetrics and Gynecology, The George Washington University School of Medicine Washington, DC, United States. *

**Keywords:** Cervical cancer, cervical lesions- HIV, visual inspection with Acetic Acid, Cambodia

## Abstract

**Introduction:**

There is a high burden of cervical cancer in Cambodia, yet published data on the prevalence of cervical dysplasia and the risk factors contributing to the development of pre-cancerous lesions in Cambodian women is very limited. In addition, as it is well known that HIV positivity increases cervical cancer risk, it is important to quantify the prevalence of cervical dysplasia and carcinoma among Cambodian women living with HIV disease.

**Methods:**

A cross-sectional study was conducted with a sample of 499 HIV+ and 501 HIV- Cambodian women at the Sihanouk Hospital Center of HOPE. Visual inspection with 5% acetic acid was the method of screening. Colposcopy was performed on all VIA+ patients, and subsequent treatment followed WHO guidelines. Logistic regression models, stratified by both HIV+ and HIV- groups, were used to assess significant factors associated with having dysplasia.

**Results:**

VIA+ results were prevalent in both the HIV+ and HIV- arms of the study. The HIV+ patients were more likely to have a lower age at coitarche, lower weight, 2 or more lifetime sexual partners, two or greater pregnancies, or be unmarried. The estimated prevalence of VIA detected cervical dysplasia was 11% for the entire study sample, 13.4% in the HIV positive (HIV+) group and 8.6% in the HIV negative (HIV-) group (OR: 1.65; 95% CI: 1.10, 2.48; p=0.01). For the HIV+ group, having a history of 4 or more full-term pregnancies (OR: 3.42; 95% CI: 1.01-11.64; p=0.049) was found to be significantly associated with having an increased risk of developing cervical dysplasia in the multivariate model.

**Conclusion:**

Cervical dysplasia is prevalent in both HIV positive and negative Cambodian women and a VIA based national screening programs need to be developed and expanded to provide access to affordable and effective treatment for cervical dysplasia and cancers.

## Introduction

Cervical cancer is highly preventable if appropriate screening is provided. In addition, even invasive carcinoma can be effectively managed if it detected at an early stage and managed appropriately (WHO, 2012). The International Agency for Cancer Research (IARC) estimates that if all women 35-64 are screened every 5 years and treated if necessary, the incidence of cervical cancer worldwide would be reduced by 84% (Ferlay et al., 2015; Blumenthal and McIntosh, 2005). Globally cervical cancer is the 4th most prevalent cancer in women, accounting for 7.5% of all cancer-associated deaths (Denny, 2015; Ferlay et al., 2015). The highest burden is observed in low- and middle-income regions of the world with Southeast Asia having the second highest burden of disease globally. In 2012, Southeast Asia reported 175,000 cervical cancer cases and 94,000 cervical deaths. In addition, Cambodia reported one of the highest cervical cancer morbidity and mortality rates in this region and cervical cancer is the most common malignancy in Cambodian women (Forouzanfar et al., 2011; Denny, 2012; Ferlay et al., 2015). 

The high rate of cervical cancer reported in Cambodia, like in other low and middle-income settings, is largely attributed to limitations in access to routine cervical dysplasia/cancer screening. While plans are underway to develop a national screen-and-treat program for Cambodian women, prevention and treatment services are currently not widely available. Barriers in access to screening include economic constraints, limited knowledge about disease risk, and the limited number of trained cervical cancer screening providers (Denny, 2015). Aside from these barriers, the most important risk factors associated with having cervical dysplasia in Cambodian women is not known. 

In recent years, Cambodia has experienced a dramatic decline in its HIV infection rates and the prevalence of HIV in Cambodia, is relatively low (Vun et al., 2014; Pe et al., 2015). However, there are still approximately 39,000 females, 15 years and older, living with HIV in Cambodia. In addition, a cervical cancer diagnosis is considered to be AIDS defining in a HIV+ woman. Furthermore, the risk of developing cervical dysplasia and invasive cervical cancer is significantly higher in women living with HIV disease and the rate of progression of precancerous cervical lesions to invasive disease is faster in this high-risk group compared to HIV- females (Maiman et al., 1997; Orem et al., 2004; Massad et al., 2015). 

As logistical and economic issues make traditional cytology-based cervical cancer screening challenging in resource-limited countries such as Cambodia, alternative screening strategies have been developed. One such strategy, visual inspection with acetic acid (VIA) is suitable for low-resource settings because it is inexpensive and does not require difficult-to-obtain materials (Lim et al., 2011; Sahasrabuddhe et al., 2012). VIA involves direct visualization of the cervix for the presence of pre-cancerous cervical lesion after the application of acetic acid 3-5% (Sankaranarayanan et al., 2004). As the results of the VIA are immediate, a women’s health specialist may treat most pre-cancerous lesions with cryotherapy in the same visit. This “screen-and-treat” strategy has been shown to be relatively easy to implement and very effective in reducing cervical cancer in other low-resource settings (Nuranna et al., 2012; Ramogola-Masire et al., 2012). 

As previously mention, there are very limited data regarding prevalence of cervical dysplasia in Cambodia. As such, the primary aim of this study is to determine the prevalence of VIA-detected cervical dysplasia in women with, and without, HIV disease. The availability of these data would inform the allocation of resources and aid in the development of relevant national programs, such as effective screen-and-treat programs, to adequately address cervical dysplasia and carcinoma in Cambodia. Additionally, this study was designed to see if any additional risk factors might increase the risk of developing cervical dysplasia in Cambodian women so that limited resources may be focused on the most at risk women.

## Materials and Methods

*Study Location and Duration*

This study was conducted from October 2014 to September 2015 at the Sihanouk Hospital Center of Hope (SHCH) in Phnom Penh, Cambodia and was approved by the National Ethics Committee for Health Research in Cambodia. SHCH was founded in 1996 with a dual mission to train medical professionals and provide healthcare to the poor and vulnerable populations in the country. Despite being located in the Cambodian capital, due to its extensive outreach programs, SCHC services women from both rural and urban areas throughout Cambodia. As such, the convenience sample of study participants used in this study represents a cross-section of Cambodian women.

*Study Population*

The study population was one thousand 30-49 year-old, non-pregnant Cambodian women. Study enrollment was stratified by the participant’s HIV status at the time of enrollment. If, at enrollment, a given participant’s HIV status was unknown, a rapid diagnostic test was conducted to determine their HIV status. Following enrollment, a physician-administered questionnaire captured demographic, behavioral and biological characteristics and information including parity, age, age at coitarche, lifetime number of sexual partners, hormonal contraceptive use, history of smoking, current marital status and cervical cancer screening history. For HIV+ participants, data on CD4 cell count, use of highly active antiretroviral therapy (HAART) and number of months on HAART was obtained from available patient medical records.

*VIA Screening and Treatment*

The lead physician (ST) assisted by another physician or a nurse conducted the VIA screening for this study. VIA was performed and interpreted based on the recommendations of the World Health Organization (WHO, 2013) the International Agency on Cancer Research and the Cambodian Ministry of Health in its standard operating procedures manual. During the same clinic visit, and after VIA screening had been performed, VIA+ participants were treated with cryotherapy. For participants who tested VIA+ and who had cervical lesions ineligible for treatments with cryotherapy (based on WHO guidelines), or who a suspected invasive cervical cancer, colposcopy and biopsy were performed at the clinic visit to aid in further evaluation and diagnosis of the observed cervical lesions. Based on the biopsy results, treatment in the form of a loop electrosurgical excision procedure (LEEP) for cervical intraepithelial neoplasia 2 or 3 (CIN2/CIN3) or radical hysterectomy in more advanced cases was subsequently performed. Collected data were entered into a secure electronic database (EpiData) by trained data entry personnel. As part of quality control of the stored data, a random 10% of the study sample was independently examined for accuracy and completeness.

**Table 1:** Characteristics of the Study Population at Enrollment, by Participant HIV Status

Descriptor	Total Population (N=1,000)	HIV- (N=501)	HIV+ (N=499)	p-value
Age, mean (SD)	39.23 (5.78)	39.23 (6.24)	39.22 (5.28)	0.97
Age at Sexual Debut,	21.58 (4.57)	22.29 (4.65)	20.87 (4.39)	<0.001
mean (SD)				
Weight, mean (SD)	53.77 (8.68)	55.42 (9.06)	52.11 (7.95)	<0.001
CD4 Cell Count, mean (SD)	--	--	508.04 (210.16)	--
Sexual Partners,				
Lifetime number (%)				
1	699 (70%)	439 (87.8%)	260 (52.1%)	<0.001
2+	300 (30%)	61(12.2%)	239 (47.9%)	
Full Term Pregnancies				
0	99 (9.9%)	37 (7.4%)	62 (12.4%)	<0.001
1	220 (22.0%)	80 (16.0%)	140 (28.1%)	
2	305 (30.5%)	158 (31.5%)	147 (29.5%)	
3	214 (21.4%)	131 (26.2%)	83 (16.6%)	
4+	162 (15.2%)	95 (19.0%)	67 (13.4%)	
Smoking, n (%)				
No	992 (99.2%)	499 (99.6%)	493 (98.8%)	0.18
Yes	8 (0.8%)	2 (0.4%)	6 (1.2%)	
Marital Status, n (%)				
Married	802 (80.2%)	442 (88.2%)	360 (72.1%)	<0.001
Divorced	96 (9.6%)	47 (9.4%)	49 (9.8%)	
Widowed	102 (10.2%)	12 (2.4%)	90 (18.0%)	
Previous Screen, n (%)				
No	547 (54.7%)	325 (64.9%)	222 (44.5%)	<0.001
Yes	442 (44.2%)	170 (33.9%)	272 (54.5%)	
Unknown	11 (1.1%)	6 (1.2%)	5 (1.0%)	
Family History				
No	942 (94.2%)	478 (95.4%)	464 (93.0%)	0.03
Yes	36 (3.6%)	18 (3.6%)	18 (3.6%)	
Unknown	22 (2.2%)	5 (1.0%)	17 (3.4%)	
Hormonal Contraception, n (%)				
No	888 (88.8%)	436 (87%)	452 (90.6%)	0.07
Yes	112 (11.2%)	65 (13%)	47 (9.4%)	
HAART Use, n (%)				
No	--	--	21 (4.2%)	--
Yes	--	--	476 (95.4%)	
Unknown	--	--	2 (0.4%)	
HAART (Number of Months), mean (SD)	--	--	72.73 (37.57)	--

**Table 2:** Estimated Prevalence of VIA Detected Cervical Dysplasia By HIV Status

Abnormality Type	Total Population (N=1,000)		HIV- (N=501)		HIV+ (N=499)	
	N (%)	95% CI	N (%)	95% CI	N (%)	95% CI
VIA positive	110 (11.0%)	(9.1, 12.9)	43 (8.6%)	(6.2, 11.3)	67 (13.4%)	(10.6, 16.7)
Cryotherapy eligible	100 (10.0%)	(8.1, 11.9)	39 (7.8%)	(5.4, 10.1)	61 (12.2%)	(9.4, 15.1)
Suspicious cancer*	10 (1.0%)	(0.4, 1.6)	4 (0.8%)	(0.2, 2.0)	6 (1.2%)	(0.3, 2.2)

*Result, HGSIL; suspect cancer

**Table 3:** Risk Factors Associated with Having Cervical Abnormalit

	Total Population (N=1000)			
Risk Factor	OR (95% CI)	p-value	aOR (95% CI)	p-value
Age	0.97 (0.94-1.01)	0.12	0.96 (0.92-0.995)	0.03
Age at Sexual Debut	0.94 (0.89-0.99)	0.02	0.98 (0.92-1.03)	0.42
Weight	1.00 (0.98-1.02)	1	1.01 (0.98-1.03)	0.6
HIV Status				
Negative (-)	1.0 (ref)		1.0 (ref)	
Positive (+)	1.65 (1.10-2.47)	0.02	1.31 (0.80-2.16)	0.28
Sexual Partners,				
Lifetime number				
1	1.0 (ref)		1.0 (ref)	
2+	1.80 (1.20-2.70)	0.005	1.62 (0.99-2.64)	0.054
Full Term Pregnancies				
0	1.0 (ref)		1.0 (ref)	
1	2.17 (0.87-5.43)	0.1	2.30 (0.91-5.84)	0.08
2	1.88 (0.76-4.63)	0.17	2.37 (0.93-6.04)	0.07
3	1.69 (0.66-4.32)	0.28	2.25 (0.83-6.11)	0.11
4+	2.58 (1.01-6.59)	0.046	3.71 (1.33-10.35)	0.01
Smoking				
No	1.0 (ref)		1.0 (ref)	
Yes	1.16 (0.14-9.48)	0.89	0.82 (0.10-6.90)	0.85
Marital Status				
Married	1.0 (ref)		1.0 (ref)	
Divorced	0.85 (0.41-1.75)	0.66	0.98 (0.46-2.07)	0.95
Widowed	1.31 (0.71-2.39)	0.39	1.64 (0.81-3.30)	0.17
Previous Screen				
No	1.0 (ref)		1.0 (ref)	
Yes	1.01 (0.68-1.51)	0.95	1.01 (0.66-1.53)	0.97
Unknown	0.90 (0.11-7.24)	0.92	0.86 (0.10-7.16)	0.88
Family History				
No	1.0 (ref)		1.0 (ref)	
Yes	1.33 (0.51-3.49)	0.57	1.49 (0.56-4.10)	0.43
Unknown	1.30 (0.38-4.47)	0.68	1.31 (0.37-4.66)	0.68
Hormonal Contraception				
No	1.0 (ref)		1.0 (ref)	
Yes	0.59 (0.28-1.25)	0.17	0.53 (0.24-1.15)	0.11

Unadjusted Odds Ratios (OR); Adjusted Odds Ratio (aOR), p-value< 0.05

**Table 4:** Risk Factors Associated with Having Cervical Abnormality By HIV Status

Risk Factor	HIV- (N=501)				HIV+ (N=499)			
	OR (95% CI)	p-value	aOR	p-value	OR (95% CI)	p-value	aOR (95% CI)	p-value
Age	0.97 (0.92-1.02)	0.19	0.96 (0.90-1.02)	0.18	0.98 (0.93-1.03)	0.36	0.97 (0.91-1.03)	0.25
Age at Sexual Debut	0.97 (0.90-1.04)	0.33	0.98 (0.90-1.06)	0.59	0.93 (0.87-1.001)	0.053	0.97 (0.89-1.05)	0.38
Weight	1.02 (0.99-1.06)	0.22	1.02 (0.99-1.06)	0.18	0.99 (0.96-1.03)	0.63	0.99 (0.95-1.02)	0.47
Sexual Partners,								
Lifetime number								
1	1.0 (ref)		1.0 (ref)		1.0 (ref)		1.0 (ref)	
2+	1.45 (0.62-3.42)	0.4	1.28 (0.50-3.24)	0.61	1.62 (0.96-2.72)	0.07	1.71 (0.93-3.15)	0.09
Full Term Pregnancies								
0	1.0 (ref)		1.0 (ref)		1.0 (ref)		1.0 (ref)	
1	5.74 (0.71-46.23)	0.1	6.11 (0.73-50.97)	0.09	1.47 (0.51-4.21)	0.47	1.57 (0.54-4.62)	0.41
2	3.50 (0.45-27.50)	0.23	3.25 (0.40-28.99)	0.28	1.69 (0.60-4.76)	0.32	2.07 (0.71-6.05)	0.19
3	2.03 (0.24-17.07)	0.51	1.94 (0.21-18.03)	0.56	2.31 (0.79-6.81)	0.13	2.60 (0.82-8.18)	0.1
4+	4.29 (0.53-34.73)	0.17	4.63 (0.50-42.95)	0.18	2.74 (0.92-8.23)	0.07	3.42 (1.01-11.64)	0.049
Smoking								
No	1.0 (ref)		1.0 (ref)		1.0 (ref)		1.0 (ref)	
Yes	<0.0001 (<0.001->999.99)	0.99	<0.0001 (<0.001->999.99)	0.99	1.29 (0.15-11.25)	0.82	0.87 (0.10-8.00)	0.9
Marital Status								
Married	1.0 (ref)		1.0 (ref)		1.0 (ref)		1.0 (ref)	
Divorced	0.70 (0.21-2.37)	0.57	0.81 (0.23-2.94)	0.75	0.91 (0.37-2.25)	0.83	1.10 (0.42-2.88)	0.85
Widowed	0.94 (0.12-7.45)	0.95	1.40 (0.16-12.19)	0.76	1.10 (0.57-2.13)	0.78	1.72 (0.77-3.83)	0.19
Previous Screen								
No	1.0 (ref)		1.0 (ref)		1.0 (ref)		1.0 (ref)	
Yes	1.28 (0.67-2.43)	0.45	1.43 (0.73-2.81)	0.29	0.74 (0.44-1.24)	0.25	0.93 (0.51-1.70)	0.82
Unknown	<0.0001 (<0.001->999.99)	0.99	<0.0001 (<0.001->999.99)	0.99	<0.0001 (<0.001->999.99)	0.99	<0.0001 (<0.001->999.99)	0.97
Family History								
No	1.0 (ref)		1.0 (ref)		1.0 (ref)		1.0 (ref)	
Yes	3.21 (1.01-10.22)	0.049	4.31 (1.24-14.87)	0.02	0.37 (0.1-2.83)	0.34		0.44
Unknown	<0.0001 (<0.001->999.99)	0.99	<0.0001 (<0.001->999.99)	0.99	<0.0001 (<0.001->999.99)	0.99	<0.0001 (<0.001->999.99)	0.65
Hormonal Contraception								
No	1.0 (ref)		1.0 (ref)		1.0 (ref)		1.0 (ref)	
Yes	0.87 (0.33-2.30)	0.78	0.66 (0.24-1.84)	0.43	0.41 (0.13-1.37)	0.15	0.38 (0.11-1.33)	0.13
Number Months on HAART	--	--	--	--	0.997 (0.99-1.003)	0.33	0.997 (0.99-1.01)	0.52
CD4 Cell Count	--	--	--	--	1.00 (0.999-1.001)	0.87	1.00 (0.999-1.002)	0.56

Unadjusted Odds Ratios (OR); Adjusted Odds Ratio (aOR); p-value< 0.05

*Statistical Analysis*

Descriptive statistics including means with associated standard deviations (SD) for continuous variables and proportions for categorical variables were calculated to describe the characteristics of the study participants. Estimation of descriptors was done first for the entire study sample and then by HIV status for the two subgroups. Differences in baseline characteristics between the HIV+ and HIV- subgroups were evaluated using t-test for continuous and chi-square test for categorical variables. 

The prevalence of all VIA detected cervical dysplasia was calculated for the entire study sample and by the participants’ HIV status. To determine the significant demographic, behavioral and biological factors associated with having a cervical dysplasia/carcinoma, univariate and multivariate logistic regression models were run using covariates listed in Table 1. Risk factors included in the final multivariate model were selected a priori and this final multivariate model was run using a p-value of 0.05. All statistical analyses of this study were conducted using SAS 9.4 (SAS Institute, Inc). 

## Results

*Participant Characteristics*

A total of 1,000 participants were recruited into the study, of which 499 were HIV+ and 501 were HIV-. The mean age of participants was 39 years (SD±5.78), and the mean age at coitarche was 21.58 years (SD±4.57). In the study sample, 54.7% of participants had never received cervical cancer screening, and 11.2% reported the use of hormonal contraceptives (Table 1). Compared to the HIV- group, the HIV+ group had a significantly lower age at coitarche, lower weight, were more likely to report having 2 or more lifetime sexual partners, were less likely to be married, and more likely to have had two or greater pregnancies (Table 1). The prevalence of VIA detected cervical dysplasia among the total study participants was 11% (Table 2). 

As expected, the prevalence of VIA detected cervical dysplasia for the HIV positive group (13.4%) was higher than that of HIV negative group (8.6%) [OR: 1.65; 95% CI: 1.10, 2.48; p=0.01] (Table 2). The prevalence of histologically confirmed high-grade dysplasia/carcinoma was 1.0% for the HIV positive group and 0.8% for the HIV negative group [OR: 1.26; 95% CI: 0.36, 4.71; p=0.75].

Two patients in the HIV- group were positive for CIN2 and 2 were positive for CIN3. No patient in the HIV- group had biopsy proven carcinoma. No patients in the HIV+ group had biopsy confirmed CIN2 but 4 had CIN3. One patient in the HIV+ group had biopsy proven invasive cervical carcinoma. 

*Predictors of VIA Detected Cervical Dysplasia*

Unadjusted and adjusted regression models were run for the entire cohort of 1,000 participants (Table 3). Participants who were HIV+ [OR:1.65; 95% CI: 1.10-2.47; p-value=0.02], had four or more pregnancies [OR: 2.58; 95% CI: 1.01-6.59; p-value=0.046], or had 2 or more sexual partners [OR: 1.80; 95% CI: 1.20-1.70; p=0.005] were more likely to have VIA detected cervical dysplasia. Age at coitarche was also significantly associated; participants with an older age less likely to have a positive result [OR: 0.94; 95% CI: 0.89-0.99; p=0.02]. All predictors became non-significant in the multivariate regression model except having 4 or more pregnancies [OR: 3.71; 95% CI: 1.33-10.35; p=0.01]. In addition, age was a significant predictor, with older participants less likely to have dysplasia [OR: 0.96; 95% CI: 0.92-0.995].

Unadjusted and adjusted regression models were run on the HIV- and HIV+ participant subgroups, with results presented in Table 4. In the HIV- group, only family history of cervical cancer was significantly associated with having VIA detected cervical dysplasia in the unadjusted model [OR: 3.21; 95% CI: 1.01-10.22; p=0.049] and adjusted multivariate model [OR: 4.31; 95% CI: 1.24-14.87; p=0.02]. In the HIV+ group none of the covariates significantly predicted having VIA detected cervical dysplasia. Having a history of 4 or more full term pregnancies was associated with having VIA detected cervical dysplasia in the adjusted multivariate model for the HIV+ group [OR: 3.42; 95% CI: 1.01-11.64; p=0.049].

## Discussion

Our findings indicate a significant prevalence of VIA detected cervical dysplasia in both HIV positive (13.4%) and HIV negative (8.6%) women in Cambodia. The prevalence of high grade dysplasia was 0.8% for HIV negative women and 1.0% for HIV positive women. In comparison, Hav et al., (2016) previously showed a 6.25% cervical dysplasia risk in HIV- negative women detected by cytology-based screening. They acknowledge, however, that a high rate of unsatisfactory pap smears in their study may have falsely lowered the detection rate of dysplasia. Rageunaud et al., (2009) reported on an exploratory cytology-based screening program of 200 HIV+ Cambodian women. This study, conducted in 2007, reported that 17% of their patients had biopsy proven cervical dysplasia/carcinoma. They also reported a high loss to follow up rate in their study and they recommended that a VIA based screening-and-treat strategy be explored for all HIV+ Cambodian women. Lastly, Lim et al reported on a study comparing VIA versus cytology based screening in 304 HIV+ Cambodian women (Lim et al., 2011). They reported that 19% of women (n=55) screened positive with VIA, however, cytology was negative for dysplasia in 67% (n=37) of these VIA+ women . Unfortunately, resource limitations prevented the authors from obtaining cervical biopsies to provide histologic confirmation of dysplasia. As such, they acknowledge that they were unable to determine the accuracy of VIA as compared to Pap smear in HIV infected women. 

All the risk factors we examined (age, smoking, number of partners, age at sexual debut, number of full term pregnancies, weight, marital status, previous cervical cancer screening, use of hormonal contraceptives, and family history) have been shown by previous studies to have a significant association with cervical abnormalities (International Collaboration of Epidemiological Studies of Cervical, 2007; Chelimo et al., 2013; Vaccarella et al., 2013). However, only family history was found to be significantly associated with the odds of having a cervical dysplasia in our HIV- group, and the 95% CI were wide. The lack of association may be due to the generally low prevalence of many of these risk factors among Cambodian women in this study as well as in the whole country (CDHS, 2014). For example, in our study, only 0.8% of participants reported a history of smoking. Also in the case of weight, the reported prevalence of obesity in the female Cambodian population is less than 3% (27) and in our study the mean weight of participants was found to be only 53.77 kg (SD±8.68). 

For the entire cohort and the HIV+ group, 4 or more full-time pregnancies were associated with - an increased risk of developing cervical dysplasia. Age at coitarche and number of partners were also associated in the univariate analysis of the entire cohort. Of course, it is likely that these risk factors are also associated with an increased risk of contracting high-risk human papilloma virus that is the causative factor in more than 99% of cervical cancer. 

Limitations of this study include the fact that 44% of study participants had had previous cervical cancer screening and appropriate treatment. As such, the rate of dysplasia in our cohort may be significantly lower than in a cohort of women who have never received screening. Secondly, it might be argued that despite SHCH’s extensive outreach into both urban and rural settings, that our convenience sample of women does not represent a true cross section of Cambodian women. Lastly, as only the VIA+ women who had lesions suspicious for high-grade dysplasia or carcinoma had confirmatory biopsies, it is possible that there were both false positives and negative VIAs. 

In conclusion, the results of our study, in combination of the aforementioned other studies, support the establishment of a national VIA based screen-and treat screening program in Cambodia. In addition, while it is important that this service be offered to all Cambodian women we recommend that a screen and treat program should ideally be integrated into pre-existing HIV services. This will ensure that HIV positive women have easy access and are frequently screened per the WHO guidelines, to allow for early detection and management of abnormalities before they progress to an advance stage. While Cambodia’s National Center for HIV/AIDS, Dermatology and STDs (NCHADS) has not yet implemented a cervical screening program for WLWH, there is momentum building for increased focus on this population if funding allows. 

*List of Abbreviations*

IARC - International Agency for Cancer Research 

VIA - Visual inspection with acetic acid 

SHCH - Sihanouk Hospital Center of Hope 

WLWH - Women living with HIV 

HAART - Highly active antiretroviral therapy 

CIN2/CIN3 - Cervical intraepithelial neoplasia 2/3 

*Trial Registration*

This study was registered with clinicaltrials.gov on 17 September 2014. The trial registration number is NCT022537.

*Declarations*

*Ethics approval and consent*

This study was approved by the National Ethics Committee for Health Research in Cambodia on 22 August 2014 (reference: 0251 NECHR). All participants gave voluntary, informed consent to participate.

## Consent for publication

Not Applicable.

## Availability of data and material

The datasets during and/or analysed during the current study available from the corresponding author on reasonable request.

## Competing interests

The authors declare that they have no competing interests.

## Funding

Financial support for this study was provided by the Bonni L. Curran Philanthropic Trust, The PECO Foundation, The Hodge and Schuyler Family Foundation and through the Sihanouk Hospital Center of HOPE’s operating funds. 

## Authors’ contributions

ST, principal investigator for the study.

SP, contributed to data analysis and manuscript preparation

CH, contributed to statistical analysis and manuscript preparation.

AT, contributed to statistical analysis and manuscript preparation 

EM, project management and manuscript preparation 

CH, data consultant, project management and manuscript preparation. 

AG, contributed to data analysis and manuscript preparation.
